# Anti-leishmanial effects of resveratrol and resveratrol nanoemulsion on *Leishmania major*

**DOI:** 10.1186/s12866-022-02455-8

**Published:** 2022-02-15

**Authors:** Parisa Mousavi, Bahman Rahimi Esboei, Maryam Pourhajibagher, Mahdi Fakhar, Zabihollah Shahmoradi, Seyed Hossein Hejazi, Hadi Hassannia, Ayatollah Nasrollahi Omran, Hamid Hasanpour

**Affiliations:** 1 Skin Diseases and Leishmaniasis Research Center, Isfahan University of Medical Sciences, Isfahan, Iran; 2 Department of Parasitology and Mycology, School of Medicine, Tonekabon Branch, Islamic Azad University, Tonekabon, Iran; 3 Dental Research Center, Dentistry Research Institute, Tehran University of Medical Sciences, Tehran, Iran; 4 Toxoplasma Research Center, Department of Parasitology, Iranian National Registry Center for Toxoplasmosis (INRCT), School of Medicine, Mazandaran University of Medical Sciences, Sari, Iran; 5 Skin Diseases and Leishmaniasis Research Center, Department of Parasitology and Mycology, School of Medicine, Isfahan University of Medial Sciences, Isfahan, Iran; 6 Immunogenetic Research Center, Faculty of Medicine and Amol Faculty of Paramedical Sciences, Mazandaran University of Medical Sciences, Sari, Iran; 7 Department of Parasitology, School of Paramedical, Ilam University of Medical Sciences, Ilam, Iran

**Keywords:** Anti-leishmanial activity, *Leishmania major*, Resveratrol, Resveratrol nanoemulsion

## Abstract

**Background:**

Leishmaniasis is a vector-borne disease that is endemic in the tropical and sub-tropical areas of the world. Low efficacy and high cytotoxicity of the current treatment regimens for leishmaniasis is one of the most important health problems. In this experimental study, anti-leishmanial effects of different concentrations of resveratrol and resveratrol nano-emulsion (RNE) were assessed.

**Methods:**

RNE was prepared using the probe ultra-sonication method. The cytotoxicity was evaluated using the MTT technique on the L929 cell line. The anti-leishmanial activities on promastigotes of leishmania were assessed using vital staining and infected BALB/c mice were used to assess the in vivo anti-leishmanial effects.

**Results:**

In vitro and in vivo assays revealed that all concentrations of resveratrol and RNE had valuable inhibitory effects against *Leishmania major* in comparison to the control group (P < 0.05). The half maximal inhibitory concentration (IC_50_) values were calculated as 16.23 and 35.71 µg/mL for resveratrol and RNE, respectively. Resveratrol and RNE showed no cytotoxicity against the L929 cell line.

**Conclusions:**

According to the potent in vitro and in vivo anti-leishmanial activity of RNE at low concentration against *L. major*, we suggest that it could be a promising anti-leishmanial therapeutic against *L. major* in the future.

## Introduction

Leishmaniasis is a tropical disease caused by a vector borne protozoan parasite of the genus *Leishmania* that is an endemic disease in 102 countries in five continents with 12 and 15 million infected cases in the world, 350 million are at risk, 1.5 to 2 million new cases and 70,000 deaths annually [1, 2]. The disease has a spectrum of clinical manifestations from self-healing skin ulcers to serious visceral manifestations. The cutaneous leishmaniasis (CL) is typically localized to the skin and infects dermal macrophages and in the more advanced stages; metastasis to the mucosal tissue and bone marrow maybe occur [2]. Iran is one of the most important endemic countries in the Middle East and all clinical forms of CL have been reported during the last decades [3–5]. CL, the most prevalent form, is caused by *L. major* and *L. tropica* in the old world, and more than 39 million people are at risk [6]. Chemotherapy is essential for leishmaniasis control and treatment. The development of drug resistance and the emergence of new strains of the parasite are the important factors that restricted the prevention and control programs and moreover, no effective vaccine has been developed yet [7]. Pentavalent antimonials, such as meglumine antimoniate (glucantime) and sodium stibogluconate (pentostam), are used as the first therapeutic line for CL [8] but due to high toxicity, high cost, long-term treatment course, drug resistance, relapse, and not significant effectiveness; new drug discovery is essential [9]. Hence, World Health Organization (WHO) recommends the use of medicinal plants and natural compounds as complementary or alternative therapies. Due to the various useful drugs derived from medicinal plants and natural compounds, access to new sources of drugs against leishmaniasis would be helpful [10–12].

Resveratrol (3,5,4' -trihydroxy-trans-stilbene) belongs to the polyphenols’ stilbenoids group, having two phenolic rings linked to each other by an ethylene bridge. This natural polyphenol has been found in at least 72 plant species, especially in grapes’ skin and seeds, and was detected in discrete amounts in red wines and various human foods [13, 14]. Resveratrol has many biological properties including antioxidant, cardioprotective, neuroprotective, antimicrobial, anti-inflammatory, and anticancer activities [15]. In addition to the mentioned biological activities, it is a phytoalexin that acts to inhibit the growth of some pathogenic microorganisms, such as bacteria and fungi. The anti-leishmanial effectiveness of the resveratrol was assessed by Ferreira et al. and Kedzierski et al. against *L. amazonensis* and *L. major* strains, respectively in an in vitro situation with the half maximal inhibitory concentration (IC_50_) of 27 and 65.5 μM, respectively [16, 17]. Resveratrol and related stilbenes in plants are contributed as an important mechanism in host defense against infection, injury, and wounding [18–20]. Nanomaterials (NMs) are advantageous as the active antimicrobial agents because of their exceedingly large relative surface to their size. Nano-sized drugs even with a low dose are high active [21]. Therefore, NMs could serve as the alternative of current anti-infective agents to control microbial infections. The aim of this study was to assess in vitro and in *vivo* activities of resveratrol and RNE on growth and proliferation of *L. major* (Iranian strain MRHO/IR/75/ER) promastigotes and BALB/c mice, respectively.

## Materials and methods

### Chemicals supply

Glacial acetic acid, Dimethyl sulfoxide (DMSO: 99%), sodium chloride, Tween 80, ethanol, and neutral red were purchased from Merck chemical company (Frankfort, Germany). Resveratrol, MTT (3-(4,5-dimethylthiazol-2-yl)2,5- diphenyltetrazolium bromide) kit, Roswell Park Memorial Institute (RPMI)-1640 medium, Dulbecco's Modified Eagles Medium (DMEM), Griess reagent (1% sulfanilamide in H_3_PO_4_ (10% v/v) in Milli-Q water), heat-inactivated fetal bovine serum (FBS), and the penicillin–streptomycin solution (10,000 U/mL) were purchased from Sigma-Aldrich (USA). Giemsa, hematoxylin, and eosin stains were obtained from Merck chemical company (Frankfort, Germany). Polyvinyl chloride (PVC) was purchased from Hatano Research Institute, Kanagawa, Japan. Amphotericin B was obtained from Abelcet, Liposome Company, Princeton, NJ (USA).

### Preparation and characterization of resveratrol nanoemulsion (RNE)

The homogenization followed by the probe ultra-sonication method was performed for preparing the nanoparticles. First, to determine the best formulation based on the percentage of lipophilic and hydrophilic surfactants, 400 mg of solid lipid with 100 mg of resveratrol and 250 mg span 80 or span 60 were melted at 85 °C. Then, the hot lipid phase was dispersed in the 1/3 aqueous solution containing 500 mg of hydrophilic surfactant (Tween 80), heated at the same temperature, homogenized by high-shear homogenizer at 13,000 rpm for 7 min (D-91126 Schwabach, Heidolph, Germany), and then sonicated by a probe sonicator (Bandelinsonopuls, 3100, Berlin, Germany) at power levels of 3.5 W for 5 min to form a coarse pre-emulsion. The mixture was then dispersed into 2/3 of an ice-cold surfactant solution preserved in an ice bath. The final mixture was sonicated again at power levels of 0.6 W for 10 min while dipped in the ice bath. This cooling phase encouraged the formation of lipid nanoparticles. The size, size distribution, zeta potential, and poly disparity index (PDI) of RNE were determined using a dynamic light scattering (DLS) technique with a Zetasizer Nano ZS (Malvern Instruments Ltd., UK) with a wavelength of 632.8 nm and scattering angle of 173°. Furthermore, the RNE size dispersion and morphology were confirmed by Transmission Electron Microscope (TEM) (Seron Technology, AIS2100, Iran).

### Preparation of *L. major*

*L. major* promastigotes, Iranian strain MRHO/IR/75/ER, were provided by the department of parasitology, Tehran University of Medical Sciences, Tehran, Iran and were cultivated in RPMI-1640 medium supplemented with 10% FBS and penicillin–streptomycin solution at 24 °C with frequent passages every three days [22].

### In vitro* assay*

The experiments were done separately for 12, 24, 48, and 72 h in a 96-well microtiter plate. At first, 100 μL RPMI-1640 medium containing 10^5^ promastigotes (at log phase) was added to each well. Then resveratrol and RNE were added separately to the wells in triplicate at concentrations of 50, 100, 200, and 400 μg/mL. Amphotericin B (20 mg/mL) as a positive control was considered in three wells, in a concentration of 12 μM. In another row, 100 µL of medium containing parasites with phosphate buffered saline (PBS) without the drug was added as the negative controls. The plate was incubated at 24 °C for 12, 24, 48, and 72 h. At the end of incubation, live and dead promastigotes were counted after adding 0.1% eosin stain with a light microscope and the IC_50_ values were calculated by nonlinear least-squares regression analysis with Microsoft Excel 2010 [22, 23].

### Assessment of the parasite load in macrophage

For the assessment of the parasite load, 2 × 10^5^ of J774.A1 macrophages cells were cultured in a 24-well microtiter plate containing a 13 mm-diameter coverslip per well, and supplemented RPMI medium supplemented with 10% FCS and 100 U/ml penicillin and 100 μg/mL streptomycin and incubated at 37 °C in CO_2_ 5% for 24 h. Promastigotes of *L. major* at the stationary phase were seeded into each well (1:10) and incubated for 24 h and the non-internalized parasites were removed by aspirating the supernatant. The infected macrophages were then treated with the concentrations of 50, 100, 200, and 400 µg/mL of resveratrol and RNE. The number of infected macrophages and the number of amastigotes per macrophage were determined by counting the number of parasites in 100 macrophages in a neubauer chamber.

### In vivo* assay*

This study was performed in accordance with ARRIVE guidelines (https://arriveguidelines.org). Female BALB/c mice (6–7 weeks old) purchased from Razi Rad Industries were used for experimentation and kept in standard boxes under controlled light and temperature conditions. Overall, 1.6 × 10^6^ promastigotes, in the stationary phase, were injected subcutaneously in the base of the tail of mice. The mice were divided into 10 groups and five mice were allocated in each group. Based on the efficacy assay in vitro, the treatment groups were as follows: groups one to five were treated with 5, 10, 15, 20, and 40 mg/kg body weight (BW) of resveratrol and groups six to eight were treated with 5, 10, and 20 mg/kg BW of RNE, respectively. Groups 9, 10, and 11 were treated with Amphotericin B (1 mg/kg BW) as the positive control and PBS as the negative control groups, respectively. After three weeks, treatment was started when local lesions were apparent. To confirm the presence of *Leishmania* parasite, samples from each lesion were smeared and fixed with absolute methanol and stained with giemsa for detection of amastigotes by using the light microscope (1000 ×). Topical treatment was used on each mouse every 28 continuous days. The diameter of lesions was measured weekly before and after treatment by dial micrometer (Starrett Dial indicator, model 25A, USA) and compared with that of untreated lesions. The ulcer size was determined according to the following formula (a: Length and b: width) [22]:

$$\mathrm{Lesion size} =\frac{\mathrm{a}+\mathrm{b}}{2}$$

### Indirectly measurement of nitric oxide (NO) production

NO levels were evaluated using Griess reaction and compared to the untreated macrophages. In a 96-well microtiter plate, 2 × 10^5^ murine peritoneal macrophages were cultured at 37 °C in 5% CO_2_ for 4 h to allow cell adhesion. 100 µL of new medium containing promastigotes in stationary phase (10 promastigotes per macrophage) was added. 100 µL of the resveratrol and RNE (50, 100, 200, and 400 µg/mL) were added to the wells with *L. major* amastigote and then incubated at 37ºC in 5% CO_2_ for 24 h. After the incubation times, the level of NO was analyzed using cell culture supernatant by an ELISA plate reader at 550 nm [24].

### Biochemical analysis

The toxic effects of prescribed treatments on the livers and kidneys of mice were assessed by analysis of the aspartate aminotransferase (AST), alanine aminotransferase (ALT), alkaline phosphatase (ALP), creatinine (Cr), urea, sodium (Na), and potassium (K) in serum samples using Pishtaz Teb kit (Iran) according to the manufacturer's instructions.

### Hemolytic assay

In vitro hemolytic activity was done by spectrophotometric method [25]. In this study, the fresh human blood samples obtained from returned unused blood bags in the blood bank (Iranian Blood Transfusion Organization) were used. A volume of 0.2 mL of the blood was mixed with 0.8 mL of the resveratrol and RNE (50, 100, 200, 400 µg/mL). The mixture was incubated for 30 min at 37 °C and then centrifuged at 1500 rpm for 10 min. The free hemoglobin in the supernatant was analyzed by UV–Vis spectrophotometer at 540 nm. The PBS and distilled water (DW) were used as minimal and maximal hemolytic controls.

### Histopathological examination

The toxicity of resveratrol (40 mg/kg BW) and RNE (20 mg/kg BW) on tissue were evaluated according to the observation of histological changes in liver, heart, and spleen of treated and untreated mice that described previously [5]. At day 28 of treatment, mice were euthanized and livers and kidneys were removed and their weights were recorded. Impression smears were prepared and fixed in 100% methanol to allow the determination of parasite burden and confirm infection. A small piece of all tissue was cut off, immersed in a 10% neutral buffered formalin solution, and processed routinely into paraffin wax. To identify histological changes, 4-μm tissue sections were stained with Hematoxylin and Eosin (H&E) and analyzed under a light microscope. The severity of histological changes was also graded as severe, moderate, or mild.

### Apoptosis assay

The promastigotes were cultured in RPMI medium supplemented with 15% heat-inactivated FBS in 96-well microtiter plate (3 × 10^5^ parasites per well) in the absence of resveratrol and RNE (as the negative control group) and in the presence of an indicated concentration of resveratrol and RNE. They were incubated at 24 °C for 24 h. Then cells were washed twice with PBS and 1 × 10^5^ cells were re-suspended in 100 μL binding buffer and incubated with 5 μL of Annexin V-FITC and propidium iodide (PI) as a counterstain for 15 min at room temperature in darkness, according to the manufacturer's instructions (BD Biosciences, San Diego, CA, USA). Finally, the percent of apoptotic and necrotic cells was evaluated by flow cytometry (Becton Dickinson) and data were analyzed using Flowjo software (version 7) [11].

### Cytotoxicity assay

L929 cell line was seeded into 96-well microtiter plates (Nunc, Roskilde, Denmark) in DMEM (100 μL) supplemented with 10% FBS and 1% penicillin–streptomycin solution. Cells then were incubated at 37 °C in a humidity of 95% and 5% CO_2_ for 24 h. Resveratrol in concentrations of 100, 200, and 400 µg/mL, RNE in concentrations of 100, 200, and 400 µg/mL, PVC, and PBS as the positive and negative controls, respectively were added to each well in triplicate. After 24 h of incubation time, 10 μL of MTT solution was added to each well. After 3 h of incubation at 37 °C, the supernatant was removed and 100 μL of DMSO was added. Finally, the absorbance at 570 nm was measured by a microtiter plate reader (BioTek ELX800, Winooski, VT, USA) and the viability was calculated by the below formula [21]:

$$\mathrm{Viability }\left(\mathrm{\%}\right)=\frac{OD Test}{OD Control}\times 100$$

### Statistical analysis

All in vitro experiments were done in triplicate. The differences between mean values of the experimental groups were done by one-way analysis of variance (ANOVA) using SPSS version 23 software (SPSS Inc., Chicago, IL, USA). A value of *P* < 0.05 was considered statistically significant.

## Results

### Characterization

**Fig. 1:**
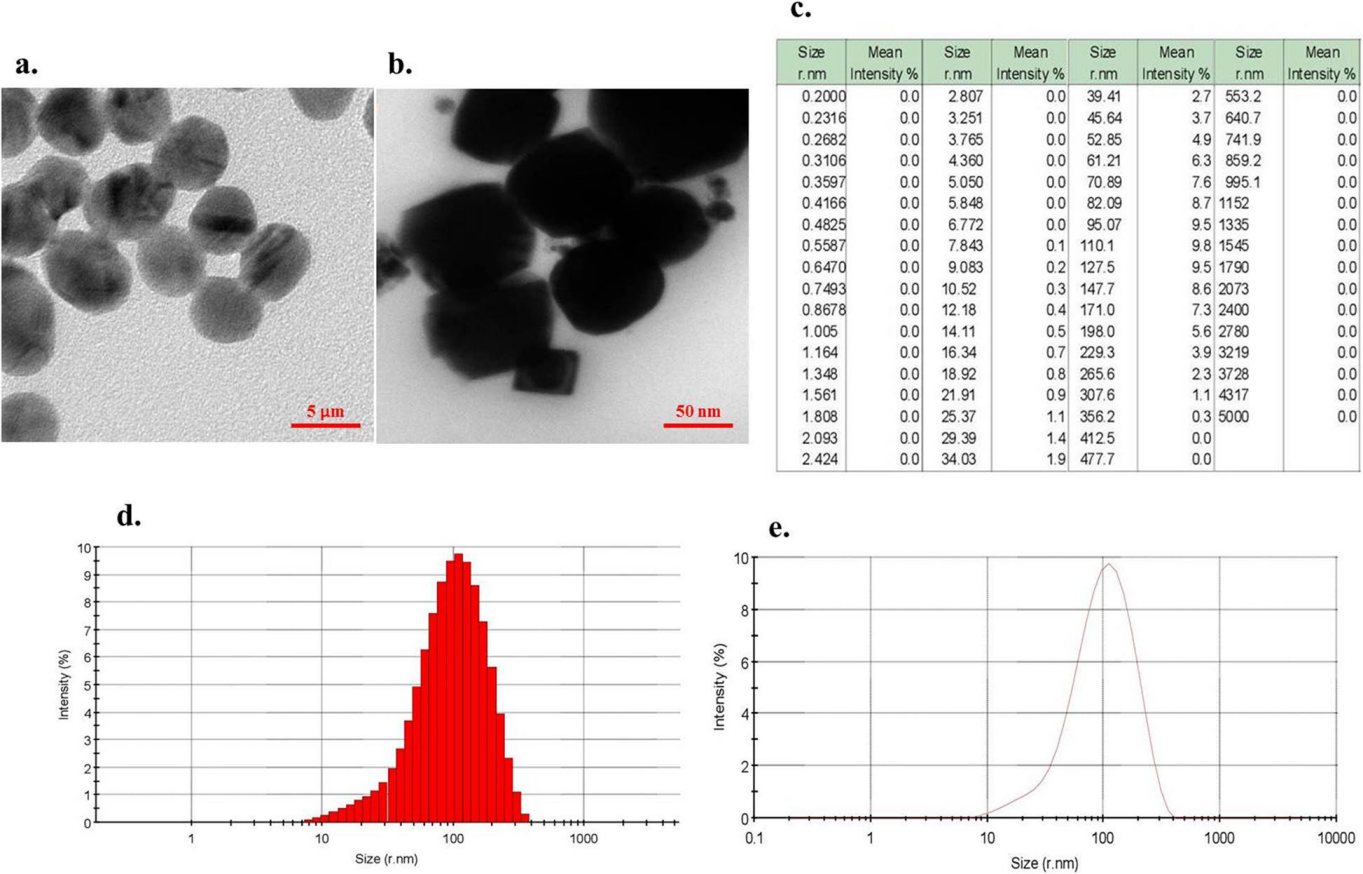
Characterization of synthesized RNE: a) Transmission electron microscope micrograph of resveratol, b) Transmission electron microscope micrograph of RNE, c) Percentages of mean intensity of RNE size, d) Histogram of size distribution of synthesized RNE, e) The particle size distribution of RNE by DLS analysis

The TEM images (Fig. 1a, b) showed that resveratrol and RNE were both spherical morphology. According to the results of DLS analysis, the average size and PDI of RNE were 110.1 ± 14 nm (with mean intensity of 9.8%; Fig. 1c-e) and 0.23, respectively. The surface charge and stability of the prepared nanoparticle were determined by zeta potential measurements.

Zeta potential is an important tool to indicate particle surface charge, which could be used to predict and control the stability of nano-suspensions. The average size and the zeta potential of resveratrol were 432.7 ± 26 nm and—12.1 mV, respectively. In contrast, the zeta potential value of RNE was found to be -48.7 mV. According to the data, the negative potential is high enough to avoid the aggregation of nanoparticles and keep their dispersion system stable.

### In vitro* activity*

**Fig. 2:**
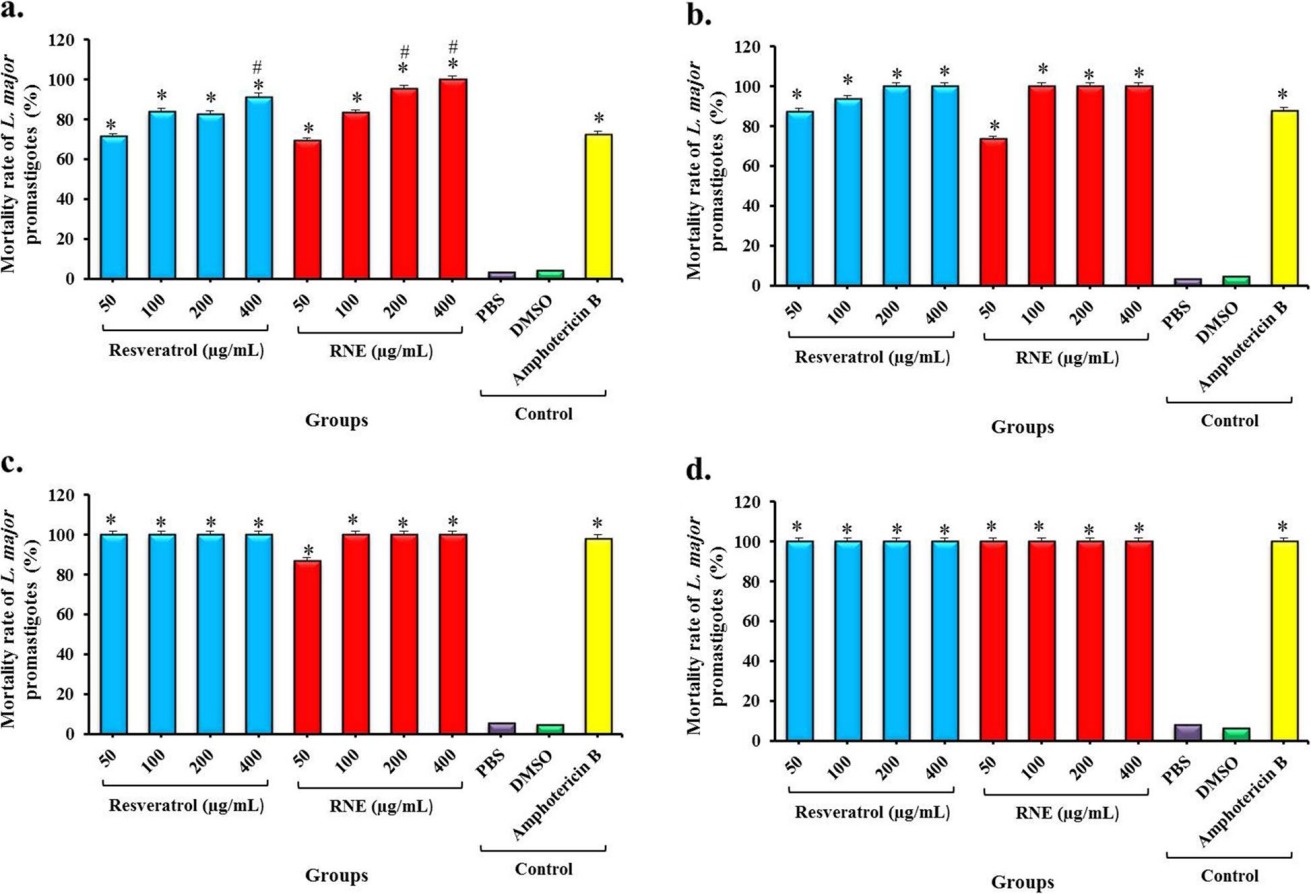
The mortality rate of *L. major* promastigotes following treatment with different groups; a) after 12 h, b) after 24 h, c) after 48 h, and d) after 72 h. *Significant differences according to the control group (PBS), *P* < 0.05. #Significant differences compared with Amphotericin B, *P* < 0.05

In all concentrations, the inhibitory effects of resveratrol and RNE were dose and time-dependent. In all concentrations by increasing the time, the amounts of live promastigotes of *L. major* were decreased. In all times, the resveratrol and RNE showed significantly better effectiveness in comparison to the negative and even positive controls. After 12 h of treatment, resveratrol and RNE at the concentrations of 50, 100, 200, and 400 µg/mL were significantly more effective than Amphotericin B (*P* < 0.05). After 24 h of treatment, resveratrol and RNE in all concentrations were significantly more effective than Amphotericin B (*P* < 0.05). Also, after 48 h of treatment, resveratrol at concentrations of 50, 100, 200, and 400 µg/mL and RNE at the concentrations of 100, 200, and 400 µg/mL showed better anti-leishmanial activity than positive control (Amphotericin B) but this difference was not statically significant (*P* > 0.05). The mortality rate of the parasite in all treatment groups after 72 h was similar to a positive control (Amphotericin B; *P* > 0.05), otherwise significantly higher than negative control group (PBS) in all exposure times (*P* < 0.05; Fig. 2).

**Fig. 3:**
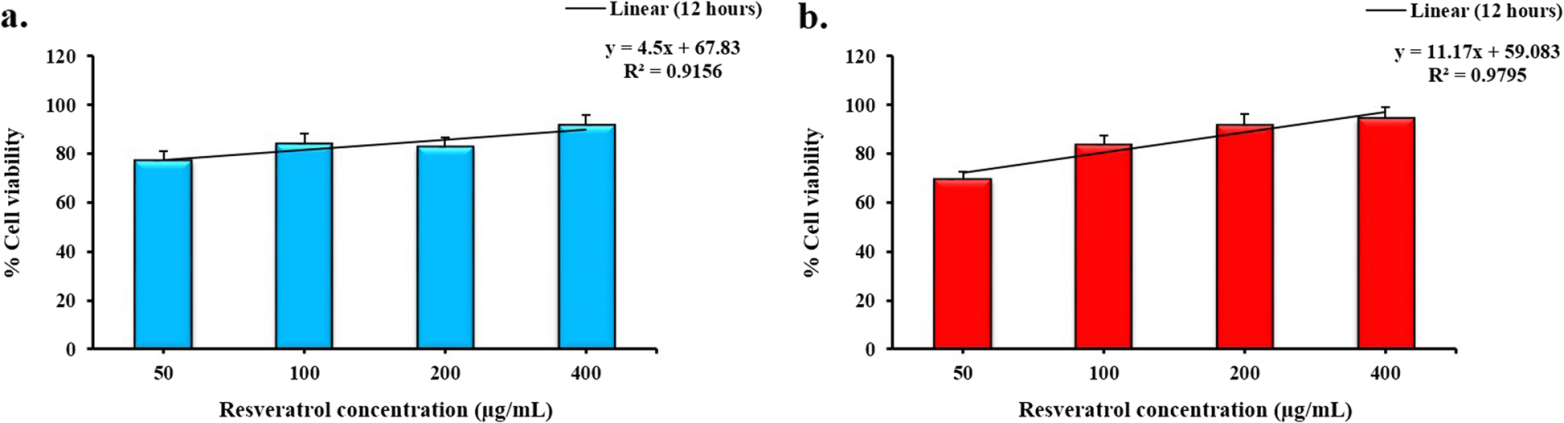
Determination of the IC_50_ value of the resveratrol (a) and RNE (b) on promastigotes of *L. major* after 12 h

Furthermore, the IC_50_ values were calculated as 16.23 and 35.71 µg/mL for resveratrol and RNE, respectively (Fig. 3).

### In vivo* activity*

**Fig. 4:**
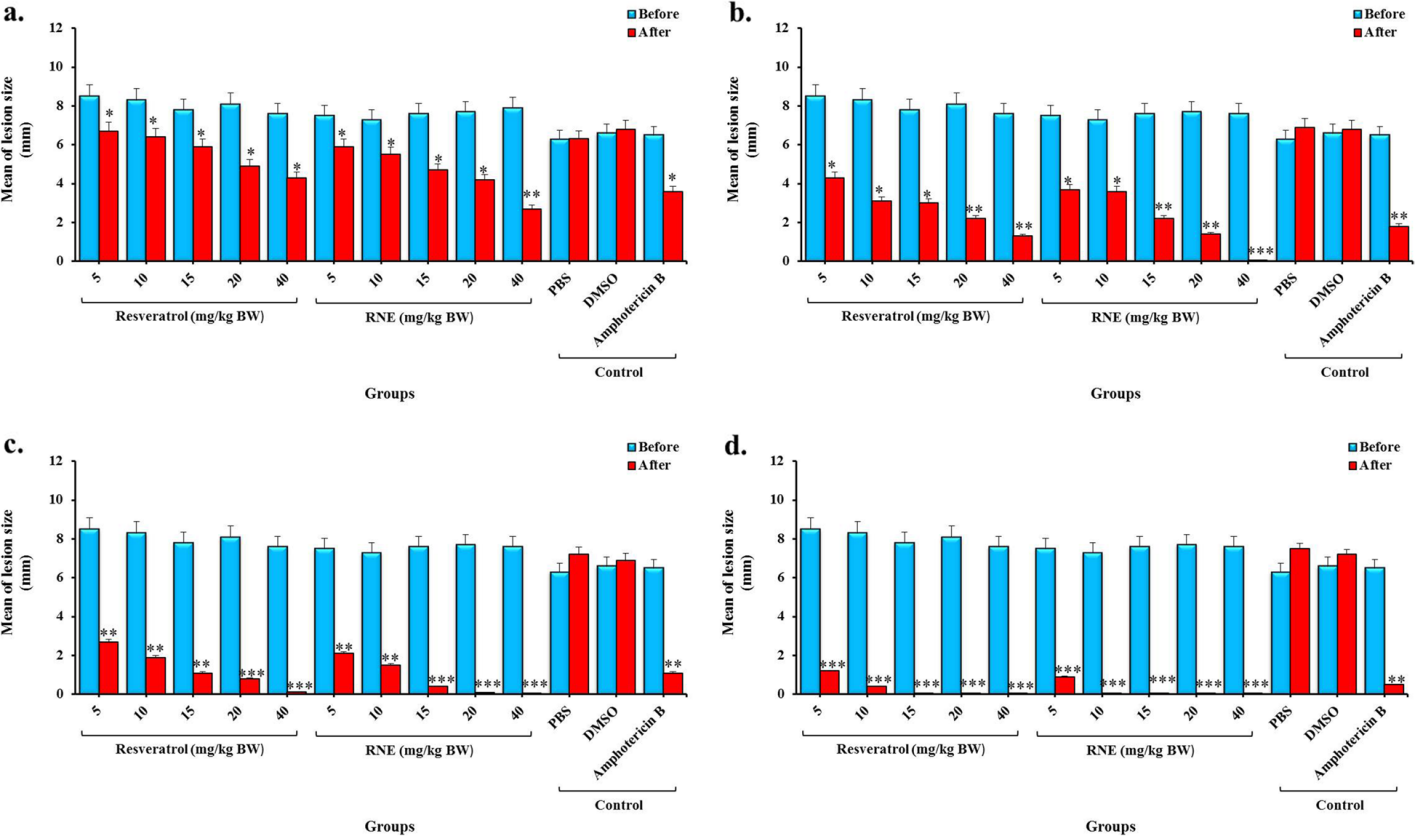
The mean of lesion size BALB/c mice following treatment with different groups; a) after 7 days, b) after 14 days, c) after 21 days, and d) after 28 days. *Significant differences according to the control group (PBS), *P* < 0.05, ** *P* < 0.005, *** *P* < 0.0005

The in vivo activities of different treatment groups were assessed for 28 days of intervention. According to the results in Fig. 4, the mean size of lesions decreased in the groups treated with resveratrol and RNE in comparison with the control group. After 7, 14, and 21 days of treatment, the differences between Amphotericin B and Resveratrol (at concentrations of 5, 10, and 15 mg/kg BW) and RNE (at concentrations of 5 and 10 mg/kg BW) were statically significant (*P* < 0.05) but, the concentrations of 20 and 40 mg/kg BW of resveratrol and the concentrations of 20 mg/kg BW of RNE showed similar efficacy to Amphotericin B (*P* > 0.05). After 28 days of treatment, all concentrations of resveratrol and RNE revealed better anti-leishmanial activity but the difference was not statically significant (*P* > 0.05). The mean lesion size in the negative control (PBS) after 28 days of intervention was 7.5 and 7.2 mm, whereas it was 0.0 mm in groups treated with the 15 mg/kg BW of resveratrol and 10 mg/kg BW of RNE. The mean difference between treated and untreated controls was significant (*P* < 0.05). There are not significant differences between resveratrol and RNE with Amphotericin B as the positive control (*P* > 0.05).

### Anti-amastigote activity

The anti-amastigote activity of Resveratrol and RNE in concentrations of 50, 100, 200, and 400 µg/mL were assessed during 24 and 48 h in comparison to the control group. Our results in Table 1 indicated that Resveratrol and RNE in all concentrations significantly decreased the rates of macrophages infectivity and the number of amastigotes in macrophages after 24 and 48 h of incubation (*P* < 0.05). Resveratrol in the concentration of 400 µg/mL and RNE in the concentrations of 200 and 400 µg/mL showed significantly better anti-amastigote activity than Amphotericin B as the positive control (*P* < 0.05). RNE in concentrations of 100, 200, and 400 µg/mL were significantly more effective than Resveratrol (*P* < 0.05) but in the concentration of 50 µg/mL there is not any significant difference (*P* > 0.05).

**Table 1:** The percentage distribution of infected macrophages and mean number of amastigotes in each macrophage in the different experimental groups after 24 and 48 h

Groups	Concentration	MQ^a^ infectivity (%)	Number of amastigotes	MQ infectivity (%)	Number of amastigotes	MQ infectivity (%)	Number of amastigotes
		**0 h (Before treatment)**		**24 h**		**48 h**	
**Resveratrol**	50 µg/mL	76.4	17.6	68.4^*^	12.5	55.3	9.8^*^
	100 µg/mL	81.0	18.3	56.2^*^	12.1	41.9^*^	9.5^*^
	200 µg/mL	78.3	18.0	42.6^*^	6.7^*^	35.5^*^	2.4^*^
	400 µg/mL	79.6	16.2	31.4^*^	3.3^*^	25.8^*^	0.5^**^
**RNE**	50 µg/mL	78.4	15.8	67.8^*^	11.6^*^	53.8	6.7^*^
	100 µg/mL	87.5	17.3	54.2^*^	7.3^*^	43.0^*^	0.6^**^
	200 µg/mL	84.5	17.6	34.3^*^	2.6^*^	26.2^*^	0.0^**^
	400 µg/mL	77.0	17.0	22.5^**^	0.0^**^	17.4^**^	0.0^**^
**Amphotericin B**	20 µg/mL	73.4	15.7	34.5^*^	4.3^*^	42.7^*^	0.4^**^
**PBS**	7.23 μM	74.5	16.3	81.7	15.2	63.2	14.6
**DMSO**	6.73 μM	80.3	16.3	73.5	13.6	67.7	12.9

a: Macrophage, *Significant differences according to the control group (PBS), *P* < 0.05, ** *P* < 0.005

### Determination of NO production

**Fig. 5:**
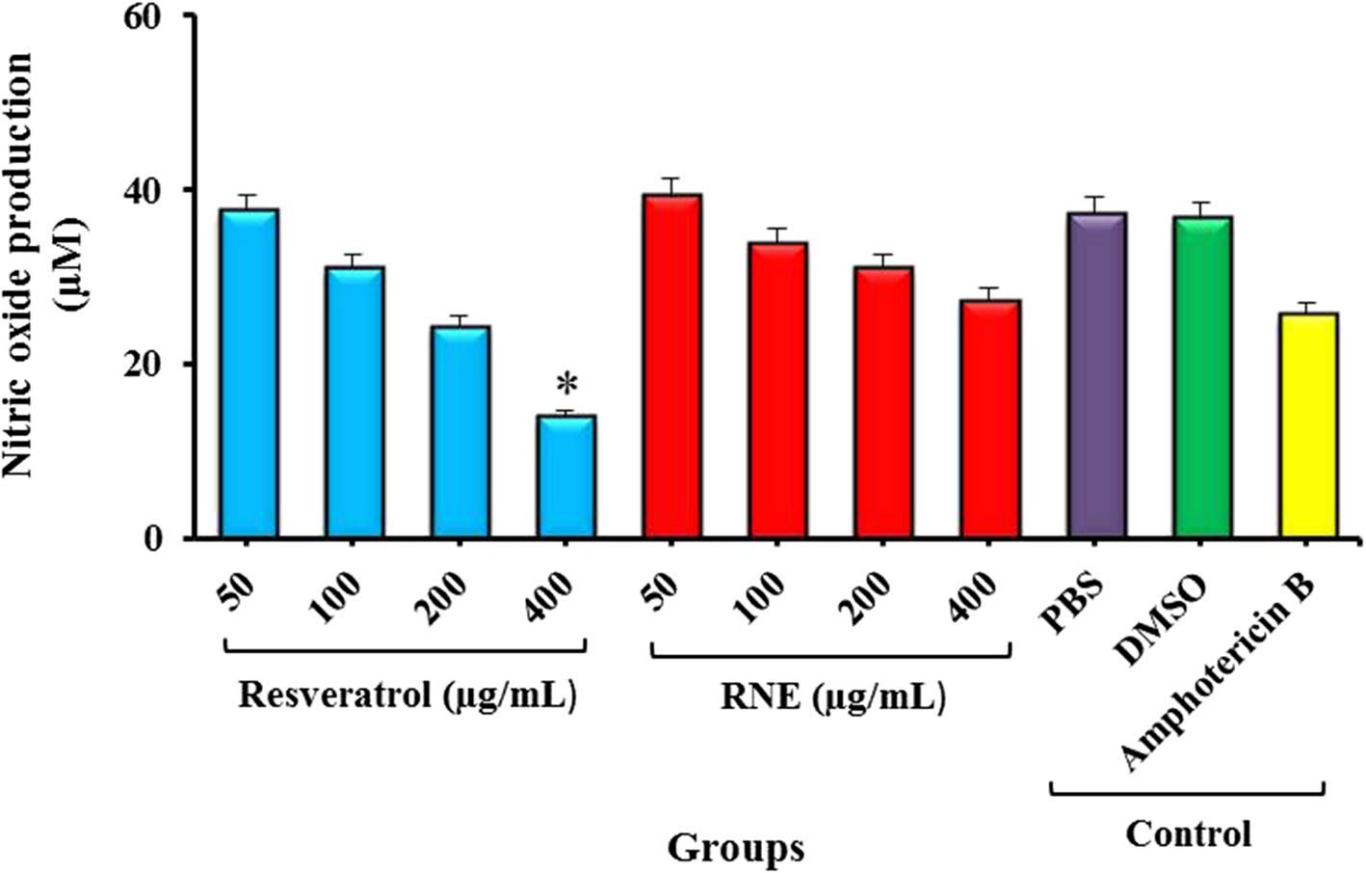
The production of nitric oxide following treatment with different groups. *Significant differences according to the control group (PBS), P < 0.05

The high concentrations of resveratrol (≥ 200 µg/mL) decreased the production of NO and produced a high lower of this component compared with the control group (PBS: 7.23 μM and DMSO: 6.73 μM). In addition, the production of NO-induced by resveratrol, RNE (at the concentrations of 200 and 400 µg/mL), and Amphotericin B were statistically different (*P* > 0.05; Fig. 5).

### Biochemical analysis

**Fig. 6:**
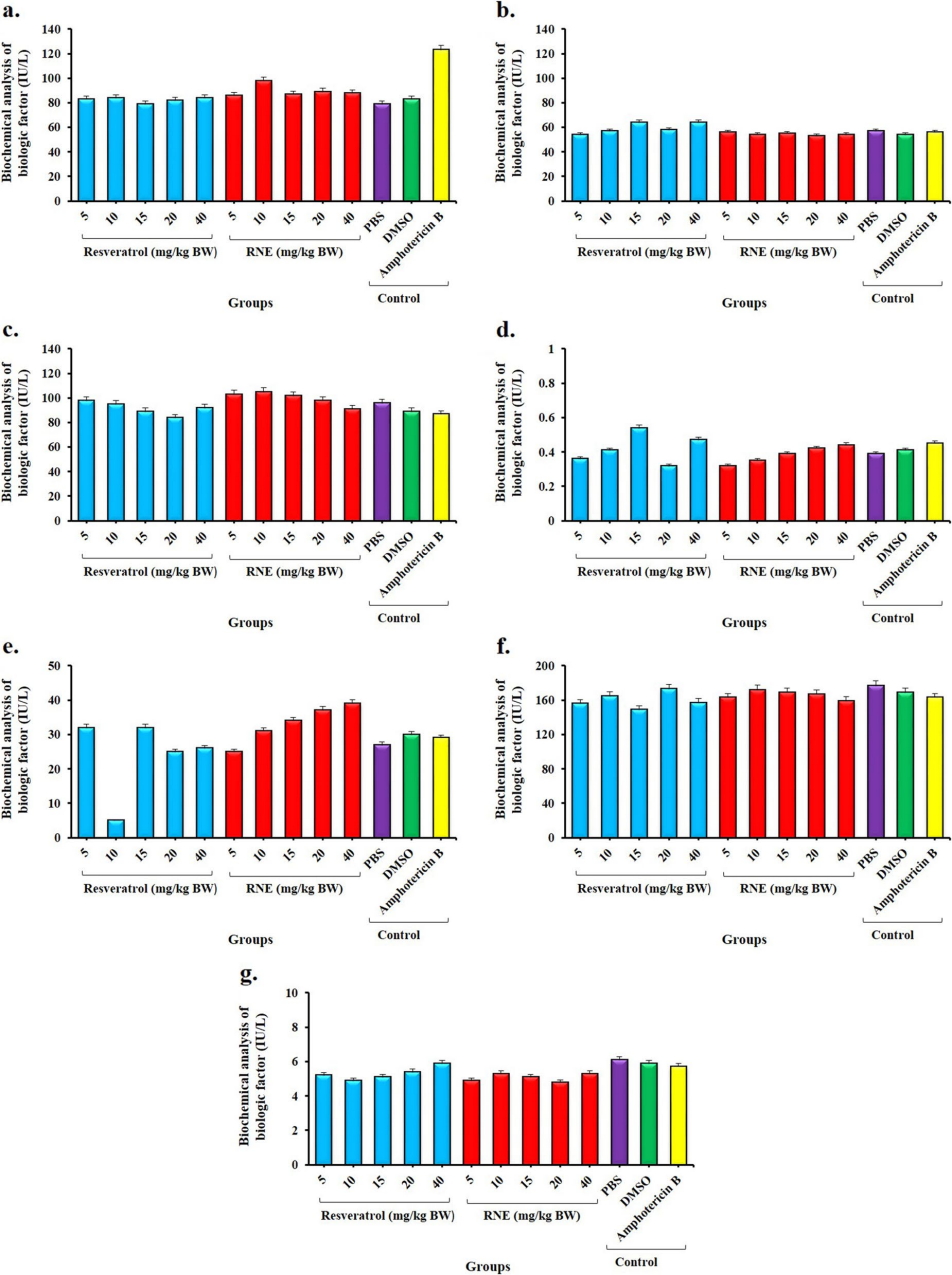
Biochemical analysis of some biologic factors in BALB/c mice following treatment with different groups; a) Aspartate aminotransferase (AST), b) Alanine aminotransferase (ALT), c) alkaline phosphatase (ALP), d) Creatinine (Cr), e) Urea, f) Sodium (Na), and g) Potassium (K). *Significant differences according to the control group, *P* < 0.05

The biochemical parameters such as AST, ALT, and ALP enzymes, Cr, urea, Na, and K were evaluated in BALB/c mice following treatment with different groups (Fig. 6a-g). No significant differences were observed between the treated and control groups (*P* > 0.05; Fig. 6), except for the AST test in group treated with Amphotericin B (Fig. 6a; *P* < 0.05).

### Hemolytic activity

According to the results in Table 2, the hemolytic effects of resveratrol and RNE in highest concentration were 25.2 ± 5.3% and 15.4 ± 3.5%, respectively, while; Amphotericin B showed 21.6% hemolytic effect compared with the control group (*P* < 0.05).

**Table 2:** The hemolytic effects of the treatment groups

Hemolytic assay	Resveratrol (µg/mL)			RNE (µg/mL)			Amphotericin B	PBS	DW^a^
	100	200	400	100	200	400			
Percent (%)	17.5 ± 2.4	19.6 ± 3.7	25.2 ± 5.3	9.4 ± 2.1	11.31 ± 2.6	15.41 ± 3.1	21.6 ± 5.3	2.3 ± 0.3	43.4 ± 7.4
*P* value	< 0.05	< 0.005	< 0.005	< 0.05	< 0.05	< 0.05	< 0.005		< 0.005

^a^*DW* Distilled water

### Histopathology findings

**Fig. 7:**
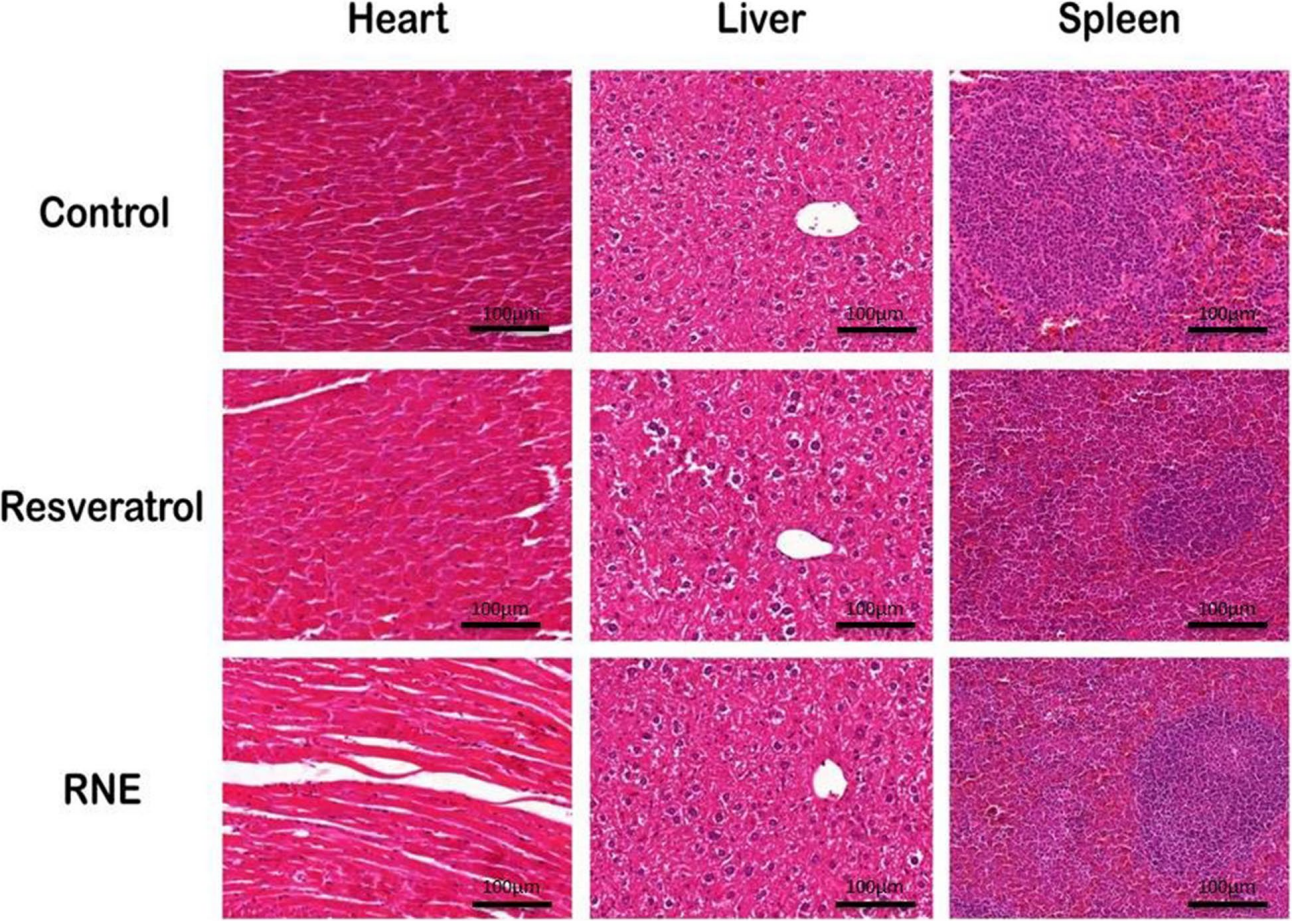
Histopathology examination of heart, liver, and spleen of BALB/c mice treated with resveratrol and RNE (40 mg/kg BW) in comparison to the non-treated control (Original magnification: × 400; Scale bar represents 100 µm)

H&E staining showed that resveratrol and RNE similarly to the control, did not cause significant pathological changes in the heart, liver, and spleen (Fig. 7).

### Flow cytometry analysis

**Fig. 8:**
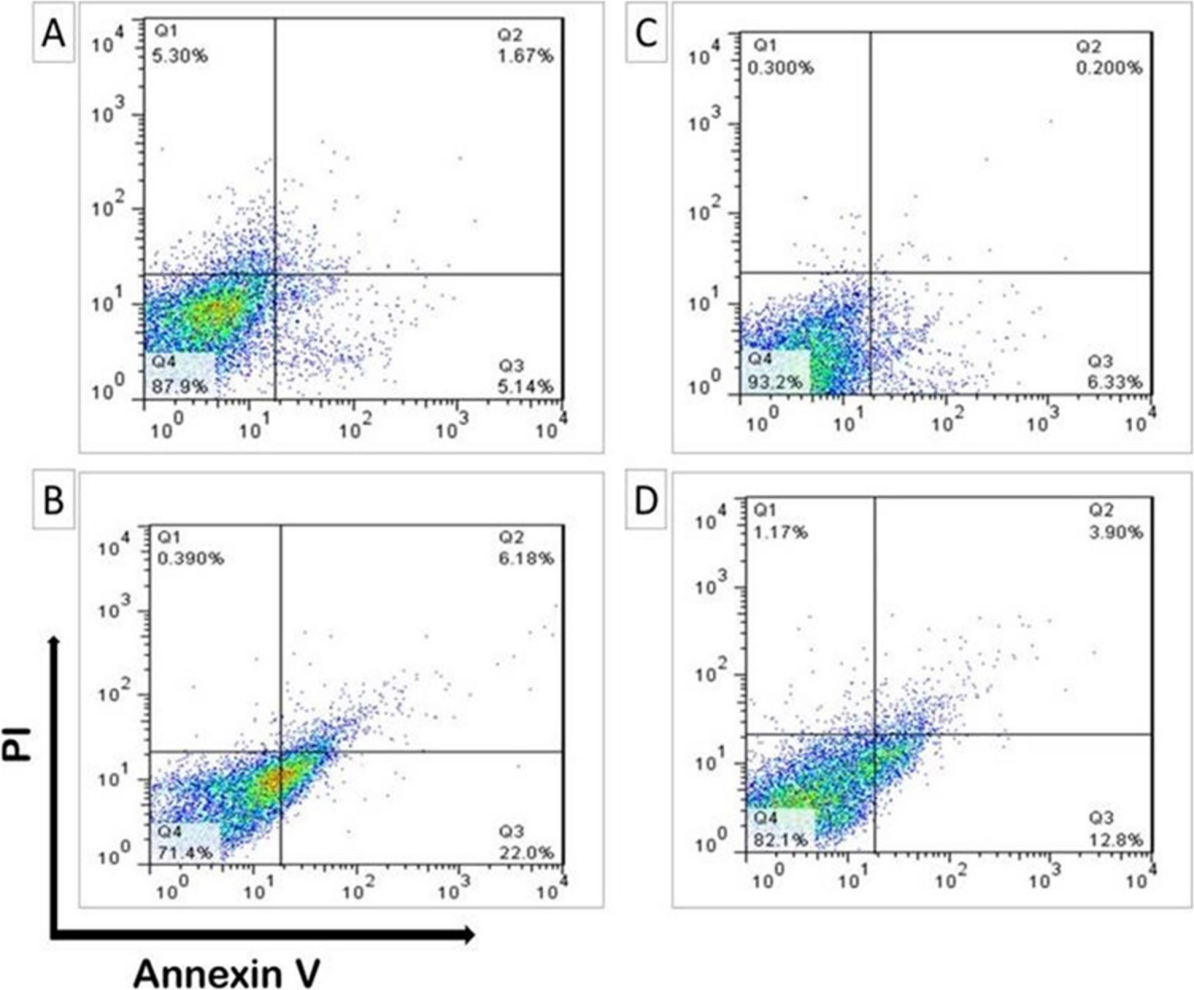
Flow cytometry analysis of treated promastigotes with Annexin V-FITC-PI staining. **A**) Treated with 200 µg/mL resveratrol, **B**) Treated with 400 µg/mL resveratrol, **C**) Treated with 200 µg/mL RNE, and **D**) Treated with 400 µg/mL RNE. Quadrant Q1, Q2, Q3, and Q4 reflect necrosis, late apoptosis, alive and early apoptosis, respectively

The treatment of promastigotes at two concentrations of resveratrol (200 and 400 µg/mL) and RNE (200 and 400 µg/mL) for 24 h resulted in necrotic and apoptotic effects in the parasites and the representative histograms of the flow cytometry (Fig. 8) showed that the viability rates of cells to be 87.9% (200 µg/mL resveratrol), 71.4% (400 µg/mL resveratrol), 93.2% (200 µg/mL RNE), and 82.1% (400 µg/mL RNE). According to the results in Fig. 8, there were no significant differences between the treatment groups (*P* > 0.05).

### Cytotoxicity activity

The tetrazolium-based colorimetric MTT assay showed that resveratrol and RNE in all concentrations had lower toxicity effects on the L929 cell line viability after 24 h in comparison to the control group (*P* > 0.05; Table 3). Furthermore, resveratrol in concentrations of 100 and 200 µg/mL and RNE in concentrations of 100, 200, and 400 µg/mL revealed lower cytotoxicity effects in comparison to the Amphotericin B as a conventional drug and these differences were statically significant (*P* < 0.05). Although resveratrol in concentration of 400 µg/mL had lower cytotoxicity effect in comparison to the Amphotericin B, it was not statically significant (*P* > 0.05).

**Table 3:** The cytotoxic effects of the treatment groups in comparison to the control groups

**Cytotoxicity assay**	**Resveratrol (µg/mL)**			**RNE (µg/mL)**			**Amphotericin B**	**PBS**	**PVC**^**a**^
	100	200	400	100	200	400			
Percent (%)	8.4 ± 1.6	14.8 ± 3.0	18.7 ± 4.6	11.4 ± 3.3	13.2 ± 2.9	15.12 ± 2.3	19.5 ± 0.6	5.3 ± 1.6	37.6 ± 5.3
*P* value	< 0.05	< 0.05	< 0.005	< 0.05	< 0.05	< 0.005	< 0.005		< 0.005

^a^Polyvinyl chloride

## Discussion

The anti-leishmanial effects of resveratrol and RNE in different concentrations were assessed in the present in vitro and in vivo study. Conventional treatments for leishmaniasis indicate too many limitations, comprising high costs, inaccessibility, high toxicity with many severe adverse side effects, and drug resistance [7, 22]. Therefore, designing accessible, low cost, and safe therapies is essentially required. Natural products due to the large structural diversity of secondary metabolites and novel chemical structures; conventionally play a crucial role in the exploration of new therapeutics and are valuable sources over centuries [26]. Resveratrol, a type of Stilbene compound, is reported as a therapeutic agent for many infectious diseases which has been extensively used in many preclinical studies [27–30].

Resveratrol can inhibit ribonucleotide reductase, cell DNA polymerase, and can disrupt the cell cycle [31]. Furthermore, several investigations have also indicated antimicrobial activities of resveratrol against different pathogens [14, 28]. For instance, Docherty et al. evaluated the effects of topically applied resveratrol on cutaneous herpes simplex virus (HSV) infections caused by an acyclovir-resistant virus in hairless mice and the results indicated that resveratrol inhibits HSV lesion formation in the skin of mice without any apparent dermal toxicity such as scaling, erythema, lichenification, crusting, and excoriation [32]. A study conducted by Jung et al. applied resveratrol to assess its antifungal effects and the results indicated that resveratrol is effective against *Candida albicans* with the IC50 value of 10–20 µg/mL with no hemolytic activity against human erythrocytes [33]. Similar results by Costa et al., reported that resveratrol at the concentration of 10 mg/mL significantly decreased the spores of *Nosema ceranae* in honey bees [34]. In some similar works resveratrol has been evaluated for its anti-parasitic activity against *Hymenolepis diminuta* [35], *Leishmania spp.* [17, 18], *Plasmodium falciparum* [36], and revealed acceptable outcomes. Kedzierski et al. investigated the effects of resveratrol on *L. major* promastigotes and intracellular amastigotes stages and the results indicated that this compound has brilliant anti- promastigote effects but the intracellular amastigote was resistant to resveratrol at concentrations of 10, 50, and 100 µg/mL [17]. In this study, the concentration of 100 µg/mL was able to eliminate 100% of promastigotes in 48 h. In a similar study conducted by Ferreira et al., resveratrol decreased the numbers of parasite in vitro, reduced the mitochondrial potential, increased the percentage of promastigotes in the sub-G0/G1 phase of the cell cycle and the nuclear magnetic resonance (NMR) spectroscopy analysis revealed that resveratrol elevated CH2-to-CH3 ratio and choline peak [16]. All these results indicated resveratrol killed the parasite. In an in vitro study by Alves Passos et al., oxyresveratrol showed anti-leishmanial effects on *L. amazonensis* promastigotes with an IC50 value of 65 µg/mL [37]. So, we could conclude that both resveratrol and RNE were more effective than oxyresveratrol and/or *L. amazonensis* promastigotes was more resistant than *L. major*. All studies examined the anti-parasitic effects in vitro while this study was conducted in an in vivo situation for the first time. Another very important point is the type of study. The fact that a drug has an extraordinary anti-parasitic effect in vitro cannot guarantee its effectiveness in vivo.

In the present study, resveratrol and RNE (50, 100, 200, and 400 µg/mL) were used for evaluation their anti-promastigote and anti-amastigote effects. Our results were compared to the Amphotericin B as the positive control and there were not any significant differences between the results of resveratrol and RNE (200 and 400 µg/mL) and Amphotericin B.

The IC50 values were calculated as 16.23 and 35.71 µg/mL for resveratrol and RNE, respectively. The most interesting point in this study was that resveratrol and RNE at the concentration of 200 and 400 µg/mL significantly indicate more effectiveness in comparison to the Amphotericin B as the conventional treatment, as well as at a concentration of 100 µg/mL also had a better effect than other studies.

Inhibition of tyrosine kinase activity in different malignancies, activation of the caspase 3 and 7, a decrease of the IL-17 production and consequently activation of the human mononuclear cells, decrease prostaglandin E2 (PGE2) production, anti-inflammatory and antioxidant activities are reported caused by resveratrol in the previous studies [38, 39].

The effectiveness of a drug will be valuable when it does not have a toxic effect. Unfortunately, conventional medications used against various pathogens have irreversible side effects. That's why the search for alternatives to these drugs and the introduction of drugs with better efficacy and less toxicity, and preferably derived from natural compounds, is one of the priorities of the health system in the world. The effects of resveratrol on leishmaniasis lesions in vivo have also been investigated and its anti-leishmanial results have been confirmed. Furthermore, the toxicity of the drug has been investigated in vivo, and the results of pathological tests have shown that the resveratrol at the concentration of 40 mg/kg BW had no toxic effect on the liver, heart, and spleen tissue of female BALB/c mice.

The production of NO by infected macrophages during down-regulation of the inducible NO synthase (iNOS) is one of the most important anti-parasitic mechanisms. The NO interacts with the NADPH-oxidase and causes cytotoxicity effects against microorganisms such as parasites in different stages of infections. In the current study, the production of the NO by macrophages was analyzed and the results indicated that resveratrol at the concentrations of 50 and 100 µg/mL and RNE at the concentrations of 50, 100, 200, and 400 µg/mL decreased the NO production even more than Amphotericin B (*P* < 0.05). Similar to our results, Alves Passos et al. revealed that piceatannol and pterostilbene decrease NO production by LPS-stimulated macrophages [37]. Furthermore, in a study conducted by Qureshi et al. resveratrol and pterostilbene inhibited the expression of TNF-α, IL-1β, IL-6, and iNOS genes in LPS-stimulated RAW 264.7 cells and LPS-stimulated peritoneal macrophages from both C57BL/6 and BALB/c mice [40].

Elgendy et al. declared that resveratrol at the concentration of 20 mg/kg BW once daily for two weeks reduces oxidative damage and inflammation in mice infected with *Trichinella spiralis* [30]. We evaluated the probably toxic effects of the drugs used in the current study by assessing the cytotoxicity on L929 cell line, hemolysis on human red blood cells, and also the biochemical parameters to find possibly toxic effects and all these results confirmed the safety of resveratrol and RNE.

Overall, the results of the current study recommended that resveratrol in vitro study at the concentrations of 25, 50, and 100 µg/mL after 72, 48 and 24 h, respectively and in vivo study at the concentration of 10 mg/kg BW after 28 days are the most effective dose and time. Also, RNE in vitro study at concentrations of 50 and 100 µg/mL after 72 and 24 h, respectively and in vivo study at concentration of 10 mg/kg BW after 28 days are the most effective dose and time.

## Conclusion

The results indicated that there were no significant differences between the anti-leishmanial effects of resveratrol and RNE. Both forms of these compounds at the concentrations of 100, 200, and 400 µg/mL showed better efficacy than Amphotericin B by both in vitro and in vivo studies. The cytotoxicity and hemolysis effects were less than Amphotericin B, no biochemical changes were observed, and all these findings declare that resveratrol and RNE are not toxic and are safe for mammalian cells. Resveratrol is delivered from natural products and also, has been commonly accessible, easily prepared, and low-priced. Therefore, resveratrol is highly recommended as a good candidate for the natural leishmanicidal agent. The results of our study recommended that resveratrol in in vitro study at the concentrations of 25, 50, and 100 µg/mL after 72, 48, and 24 h, respectively and in in vivo study at the concentration of 10 mg/kg/BW after 28 days were the most effective dose and time. Also, RNE in in vitro study at the concentrations of 50 and 100 µg/mL after 72 and 24 h, respectively, and in in vivo study at the concentration of 10 mg/kg/BW after 28 days are the most effective dose and time.

## Data Availability

All data generated or analysed during this study are included in this published article.

## Abbreviations

ALP: Alkaline phosphatase
ALT: Alanine aminotransferase
AST: Aspartate aminotransferase
CL: Cutaneous leishmaniasis
DMEM: Dulbecco's Modified Eagles Medium
RNE: Resveratrol nanoemulsion
RPMI: Roswell Park Memorial Institute
PVC: Polyvinyl chloride
